# Pregnant women and substance use: fear, stigma, and barriers to care

**DOI:** 10.1186/s40352-015-0015-5

**Published:** 2015-02-12

**Authors:** Rebecca Stone

**Affiliations:** School of Criminology and Justice Studies, University of Massachusetts Lowell, 113 Wilder St, Ste. 400 Lowell, MA, 01854 USA

## Abstract

**Background:**

Substance use during pregnancy and motherhood is both a public health and criminal justice concern. Negative health consequences associated with substance use impact both the mother and the developing fetus, and there are ongoing attempts to criminalize substance use during pregnancy that put pregnant substance-using women at risk of detection, arrest, and punishment. This study explored the experiences of substance-using mothers as they navigated health and criminal justice consequences and accessed needed resources in the community.

**Methods:**

In-depth life history interviews were conducted with 30 recently-pregnant women who had used alcohol or other drugs during their pregnancies. The three-part interview schedule included questions about past and current substance use, life history, and experiences with criminal justice authorities, child protective services, and health professionals.

**Results:**

Women’s stories highlighted their strategies for managing their risk of detection by health or criminal justice authorities, including isolating themselves from others, skipping treatment appointments, or avoiding treatment altogether. Women described multiple barriers to treatment and healthcare, including a lack of suitable treatment options and difficulty finding and enrolling in treatment.

**Conclusion:**

The findings suggest that policies that substance-using women find threatening discourage them from seeking comprehensive medical treatment during their pregnancies. The implications of the findings are discussed, particularly the need for further expansion of treatment programs and social services to meet the needs of substance-using women.

## Background

Pregnant women who misuse substances (alcohol, tobacco, and prescription and illicit drugs) are positioned at the nexus of public health and criminal justice intervention. The impact of their substance use on their personal health and the health of their fetuses is a public health concern, as professionals in this field are dedicated to improving maternal and infant health. In addition, the past three decades have seen prenatal substance use become a criminal justice issue as the fetal protectionism movement spurred the increasing use of criminal sanctions for “deviant” mothers. Substance-using pregnant women, especially women of color and women in lower socioeconomic brackets, are subject to increased surveillance and may face arrest, prosecution, conviction and/or child removal (Banwell & Bammer, 2006; Boyd, 1999; Chasnoff et al. 1990; Figdor & Kaeser, 1998; Murphy & Rosenbaum, 1999; Paltrow, 1999; Paltrow & Flavin, 2013; Roberts, 1991).

Figures from the most recently-published report from the National Survey of Drug Use and Health (Substance Abuse and Mental Health Services Administration, 2012) state that, of pregnant women aged 15–44, 9.4% reported current alcohol use, 2.6% reported binge drinking, and 0.4% reported heavy drinking. Of pregnant women aged 15–44, 17.6% report smoking tobacco in the last month, a figure that represents a small, nonsignificant increase from the 2009–2010 and 2008–2009 findings. The percentage of pregnant women in this age group who report smoking tobacco in the last month has not changed significantly in the last decade, while tobacco use among nonpregnant women in the same age group has decreased slowly but significantly each year. Of pregnant women aged 15–44, 5% report current illicit drug use, a proportion not significantly different than in the previous study year. The rate of illicit drug use varies widely with the woman’s age. Teenaged pregnant women have the highest rates of illicit drug use (15–17, 20.9%), followed by young adult women (18–25, 8.2%) and adult women (26–44, 2.2%). There are no reliable nationwide estimates of the annual number of infants born after prenatal substance exposure. For example, the CDC website reports that the rate of Fetal Alcohol Syndrome (FAS) is 0.2-1.5 cases per 1,000 live births (CDC, 2014), although this estimate appears to be based on research from the 1990s. A recent study found the rate of FAS in one Midwestern community to be 6–9 per 1,000 children, and more general Fetal Alcohol Spectrum Disorder (FASD) as high as 24–48 per 1,000 children (May et al. 2014). Some infants prenatally exposed to opioids exhibit symptoms of Neonatal Abstinence Syndrome (NAS), including hyperirritability and dysfunction of the nervous system, gastrointestinal tract, and respiratory system (Finnegan & Kaltenbach, 1992). Between 2000 and 2009, the incidence of NAS among hospital-born newborns increased from 1.20 to 3.39 per 1,000 live births per year. Total hospital charges for NAS during this time period are estimated to have increased from $190 million to $720 million, adjusted for inflation (Patrick et al. 2012). While it is possible that some of the increase in NAS diagnoses could be attributed to growing recognition of NAS symptoms and increased surveillance of pregnant women, it appears that prenatal exposure to substances is a significant public health problem.

Concerns about fetal drug exposure have given rise to new laws and applications of existing laws that seek to deter women from using substances during their pregnancies and to punish those who do. The enforcement of these laws raises questions of fetal personhood and the extent to which the government may regulate pregnant women’s bodies. Substance abuse during pregnancy is considered child abuse under civil child welfare statutes in seventeen states, and in three states (Minnesota, South Dakota, Wisconsin) it is grounds for civil commitment (Murphy, 2014). 36 states recognize embryos or fetuses as potential victims of crime, although 24 of these expressly exempt pregnant women from prosecution for causing injury to their own fetuses (Murphy, 2014). In many cases, prosecutors have used laws written to target for child abuse, child neglect, contributing to the delinquency of a minor, causing the dependency of a child, child endangerment, delivery of drugs to a minor, drug possession, assault with a deadly weapon, manslaughter, and homicide (Paltrow, 1992) despite, in some cases, the aforementioned provisions protecting pregnant women from punishment (Flavin, 2009; Paltrow & Flavin, 2013). In some states, the protection from prosecution is incomplete, e.g. in Arkansas, women are exempt from being prosecuted for the homicide of their own fetuses, but may be prosecuted for battery of their fetuses (Murphy, 2014: 865).

In South Carolina, the ruling in *Whitner v. State* affirmed the conviction of criminal child neglect for a mother whose newborn tested positive for cocaine metabolites. The South Carolina Supreme Court stated that “the plain meaning of “child” as used in [the child endangerment statute] includes a viable fetus” (*Whitner v. State*, 1997). The court reaffirmed its stance on the issue in *State v. McKnight* (2003), when it upheld Regina McKnight’s 2001 conviction for homicide by child abuse after her pregnancy ended in a stillbirth attributed to Knight’s use of crack cocaine. In 2008, the same court unanimously ruled that McKnight did not have a fair trial, recognizing that McKnight’s counsel failed to make use of existing evidence from “recent studies showing that cocaine is no more harmful to a fetus than nicotine use, poor nutrition, lack of prenatal care, or other conditions commonly associated with the urban poor” (*McKnight v. State*, 2008). McKnight was released from prison after serving more than eight years (NAPW, 2008). McKnight’s case is similar to the ongoing case of Rennie Gibbs of Mississippi, who was indicted in 2007 for “depraved heart murder” when she delivered a stillborn daughter whose blood showed traces of a cocaine byproduct. Gibbs was only 16 at the time. Experts who later examined the autopsy reports concluded that the more likely cause of death was umbilical cord compression. Murder charges against Gibbs were dropped in April, 2014, after more than seven years of legal entanglements. Charges were dismissed without prejudice, leaving the possibility for charges to be refiled (Fowler, 2014).

In 2013, Alabama became the second state to explicitly allow pregnant women who use drugs to be charged with criminal child abuse when the Supreme Court of Alabama held that a viable fetus is considered a “child” for the purposes of the state’s criminal statute prohibiting the chemical endangerment of a child (Murphy, 2014: 862). Most recently, in April, 2014, Tennessee became the first state to explicitly criminalize drug use during pregnancy through legislation. Governor Bill Haslam signed SB1391 into law in April, 2014, amending Tennessee’s fetal homicide law to allow women to be prosecuted for the use of narcotics while pregnant. The law allows women to be charged with aggravated assault, which carries a penalty of up to 15 years in prison (TN SB1391, 2014).

It is difficult to produce an accurate count of the number of such cases, as there multiple barriers to the full identification and documentation of cases that, for example, do not result in published court opinions and do not receive public attention. The most recent estimate comes from the work of Lynn Paltrow, Jeanne Flavin, and the National Advocates for Pregnant Women. Paltrow and Flavin (2013) document 413 cases from 1973–2005 in which pregnancy was “a necessary factor leading to attempted and actual deprivations of a woman’s physical liberty” (2013: 299). Illicit drug use was mentioned in 348 (84%) of these cases. This record also notes the geographic distribution of cases and the sociodemographic status of the defendants, finding that the largest percentage of cases originate in the South (56%), followed by the Midwest (22%). 59% of the cases involved women of color, and women were represented by indigent defense in 71% of cases. These figures support the argument that punitive policies regarding substance use during pregnancy are disproportionately enforced against poor women and women of color.

The authors believe that this work is a “substantial undercount” (Paltrow & Flavin, 2013: 304), noting that the lack of searchable databases of cases, the confidentiality of family and juvenile court proceedings and civil commitment proceedings, the lack of media coverage of hospital detentions and compelled treatment, and lack of access to court records from Native American tribal courts. The record is also unfortunately out of date by almost a decade and, as the above-mentioned cases, court decisions, and legislative acts demonstrate, the arrests and prosecutions of pregnant and substance-using women continue. A very recent *New York Times* opinion piece by Paltrow and Flavin (2014) updates the record, stating that “since 2005, we have identified an additional 380 cases.” The arrest and prosecution of pregnant women persists despite extraordinary consensus by public health and medical associations that such actions undermine attempts to improve maternal and infant health (e.g., American College of Obstetricians and Gynecologists, 2011; National Perinatal Association, 2011; American Psychiatric Association, 2001; American Nurses Association, 1991; American Academy of Pediatrics, 1990; March of Dimes, 1990; National Council on Alcoholism and Drug Dependence, 1990).

In some places, e.g. Tennessee, women charged with substance use during pregnancy may be allowed to use evidence of finding and attending treatment as an affirmative defense. However, pregnant women seeking substance use treatment may find that there are no suitable treatment programs available. For example, Tennessee SB 1391 allows women who give birth to substance-exposed infants to avoid charges by successfully completing a court-approved treatment program, but it is yet unknown what will be “court-approved” and what counts as “completing” a program. Specialists are concerned that methadone treatment and other opiate replacement therapies, considered the gold standard for managing opioid addictions, will not be accepted by the courts. Furthermore, the bill did not create any new treatment options, expand existing options, or provide additional funds to care for patients (Beyerstein, 2014). Journalists from “America Tonight” contacted every treatment program listed on the Tennessee Department of Health and Human Services website that claimed to treat pregnant women and found five clinics that would allow pregnant women to enroll in their program and accepted Medicaid. Two of the programs were full, leaving fewer than 50 beds available (Dosani, 2014).

In the late 1990s hospital staff at the Medical University of South Carolina (MUSC) worked with police to search pregnant patients for evidence of drug use and facilitate in-hospital arrests. Proponents of this policy claimed that the goal was to get women into treatment because they would not go voluntarily. At the time, not a single drug treatment program in the state provided services for pregnant and parenting women (Paltrow, 2002: x). Furthermore, in spite of the claim that drug-exposed children were severely harmed (and thus the justification for punishment of drug-using women), no program to treat or monitor the children existed (Paltrow et al. 2000).

Contrary to claims that arresting and prosecuting pregnant women will encourage them to desist from substance use and thus improve maternal and fetal health, fear of detection and punishment presents a significant barrier to care for mothers and pregnant women. Women have reported that they delayed or avoided prenatal care altogether out of fear of punishment (Murphy & Rosenbaum, 1999; Poland et al. 1993; Roberts & Nuru-Jeter, 2010; Roberts & Pies, 2010). The effect of stigmatization, discrimination and fear of punishment present a barrier to wanted care. This creates a health risk, since substance-using women who *do* receive prenatal care experience more positive birth outcomes and have greater opportunities for other health promoting interventions than women who do not receive care (Berenson et al. 1996; El-Mohandes et al., 2003; Green et al. 1979; MacGregor et al. 1989; Racine et al. 1993; Richardson et al. 1999). The burden of these policies falls disproportionately on poor women and women of color, as those who use public health and social services are subject to increased surveillance and heightened risk of being tested and reported to criminal justice authorities (Chasnoff et al. 1990; Paltrow & Flavin, 2013; Roberts, 1991, 1999; Woliver, 2002). Women who can afford private physicians and avoid public services are likely better able to avoid testing, detection, and reporting. The probable consequence of this disparity is a widening of the health inequality across class and race divisions.

The purpose of this study is to gain a greater understand of the way substance-using women navigate the health and justice systems in order to avoid criminal justice consequences and to access needed health and social support resources. In-depth interviews with recently pregnant women who used alcohol or other drugs while they were pregnant highlight women’s strategies for avoiding being identified as substance-users and their efforts to access substance use treatment programs. The results identify the ways in which fear and stigma create barriers to care and result in unmet needs for this population.

## Method

The current study uses qualitative methodology to provide rich description of women’s experiences in their own words. A loosely-structured interview schedule of open-ended questions helped to guide the conversation through the topics of identity, health behaviors and barriers to care. Participants were encouraged to tell their stories using their own words and narrative styles.

The target population for this study was women who were pregnant or recently pregnant (within the last twelve months) and who had used alcohol, tobacco, illicit drugs, or misused prescription or over-the-counter medications at any time during their most recent pregnancies. The targeted sample size was 30 women. The participants were selected through purposive sampling with the goal of sampling a wide range of women with different sociodemographic characteristics and substance use histories (Kerlinger & Lee, 2000; Patton, 2002). As a result, the sample is not representative of any general population, but is selected for the purpose of generating “theoretical jumping off points” (Thompson, 1999). The sample was drawn from a post-industrial Midwestern city with a population of approximately 100,000 residents.

Recruitment flyers were posted in the maternity wards of local hospitals and at drug treatment centers, community centers and service enrollment offices. Flyers posted at local transportation hubs (e.g., the central bus station) were especially productive. Examples of other recruitment locations included bathrooms at the local library, pregnancy support clinics, obstetricians’ and pediatricians’ offices, and substance abuse support group meeting locations. Women who completed interviews were also invited to refer others to the study. In fact, invitation was not usually necessary; women volunteered to pass along recruitment materials to other women they knew might like to participate. These recruitment strategies proved highly effective and all 30 interviews were completed during a five-week period. Due to the broad scope of the recruitment criteria, very few women were turned away. Those who were did not meet the criteria (e.g., had not used substances during their pregnancies) or represented recruitment targets that had already been satisfied by previous participants. Interviews were completed in a single session in a place where women felt comfortable. At the end of the interview, women received a $50 VISA giftcard.

The sample consists of 30 women between the ages of 19 and 41 (Table 1). The mean age is 28.5 years old. Slightly more than half the women in the sample self-identified their race or ethnicity as White (n = 16, 53.3%). Eight women in the sample self-identified as Black or African-American (n = 8, 26.7%), one woman identified as Hispanic (3.3%) and one woman as Native American (3.3%). Four women in the sample self-identified as “other” (13.3%). Two of these women were of mixed race, one woman was adopted and did not know the races of her parents, and the fourth preferred not to answer.

**Table 1:** **Description of sample (N = 30)**

**Variable**	**Mean/% (n) [Range]**
Age	28.5 [19–41]
Number of children	2.8 [1–8]
Race/Ethnicity	
White	50.0% (15)
African American	26.7% (8)
Hispanic	6.6% (2)
Native American	3.3% (1)
Mixed/Other	13.3% (4)
Health Insurance	
None	13.3% (4)
Health Plan	13.3% (4)
Medicaid	56.7% (17)
Private Insurance	16.7% (5)
Education	
Less than high school	23.3% (7)
GED	20.0% (6)
High school graduate	26.7% (8)
Some college	16.7% (5)
College +	13.3% (4)

The criteria for participation in the study included being currently pregnant or having recently (within the past 12 months) given birth. The current pregnancy or recent birth did not have to be the participant’s first child. The number of born children per participant ranged from one to eight $\left(\overline{x} = 2.8\right)$.

In the three months prior to discovery of the most recent pregnancy, the most common substance used was tobacco (n = 26, 86.7%). Alcohol (n = 18, 60.0%), marijuana (n = 17, 56.7%) and prescription medications (n = 16, 53.3%) were also very commonly used. Of the 16 women who reported misusing prescription medications in this time period, 12 (75% of prescription medication users) reported using opioid analgesics like Vicodin, Percocet, Dilaudid, Fentanyl and Lortab. Six women (37.5% of prescription medication users) reported misuse of prescription benzodiazepines. The most common benzodiazepines used by participants were Valium and Xanax. Women usually referred to the drug by its brand name or with the catch-all “benzos”.

Less common were other substances including cocaine, methamphetamine, heroin and hallucinogens. These substances were far more likely to appear in women’s lifetime histories of substance use than to be mentioned in the three months prior to the most recent pregnancy. For example, 14 (46.7%) women reported lifetime use of hallucinogens, but only two (6.7%) women reported using hallucinogens three months prior to discovering their recent pregnancies.

## Results

The interview recordings were manually transcribed and coded for the themes of detection-avoidance strategies and experiences accessing treatment. Several common strategies and experiences were identified. Women reported feeling fear of being identified as substance-users by medical professionals or other authorities and discussed their strategies for avoiding detection. They described how they felt that others’ perceptions of them as substance-users had influenced the type of medical care they received. Finally, women talked about their experiences of seeking treatment for their substance use, the barriers they encountered, and which types of treatment were most effective for them and why.

### Fear of detection

Twenty-two women (73.3%) reported that during their pregnancies they had been afraid of being identified as substance-users. The scenarios of which they were most afraid were testing positive for substances at prenatal visits or after delivery, losing custody of their newborns and/or their older children, and experiencing criminal justice consequences for their substance use.

The remaining eight women (26.7%) in the sample reported that they were not afraid of detection. For most of these women, this was because they were not using illegal substances. Though they recognized the harmful effects of alcohol and tobacco, they were not worried about being tested, having positive test results, losing their children or being arrested. Women who were using illegal substances but were not afraid of being detected said that they felt they had their substance use “under control” and that they could avoid detection. Some women were simply unaware that they might be tested at prenatal visits or at the hospital and that they could lose their children. For example, when asked if she had feared coming into contact with the police or Child Protective Services (CPS), Brittany said that it hadn’t really occurred to her to be afraid until it was too late:

**Interviewer**: Did you have any concerns about CPS taking the children?

**Brittany**: I guess I would say no, only because nothing like that had happened before. So I hadn’t seen it and I never really knew anybody who it had happened to, either, so it really almost didn’t even seem like a possibility, I mean. That’s why there’s kind of like a lot of like, that feeling, like the CPS thing ‘cause like I said I never knew anyone really before that it had happened to, so there’s nobody I can relate with or, you know, hear about how it went or it worked out with them or anything like that, so.

Brittany had permanently lost custody of her three boys. She had managed to keep her opioid addiction a secret for many years until it spiraled out of control. She spoke about how her addiction had never resulted in contact with the police because it was her boyfriend who would take risks and go out to buy their heroin. “So I guess I was kinda sheltered in that way,” she reflected, “We were a using couple, so I guess that’s different than doing it yourself”.

Women who were using illegal substances and did not feel afraid of being identified as substance users were the exception. Pregnancy was a time of great uncertainty for most of the women, and this was compounded by the threat of detection. This was especially true for women who did not know what to expect at prenatal appointments or delivery. Some women believed they were drug-tested at every prenatal visit and that every baby delivered at the hospital had his or her meconium tested for drugs. Other women felt that the decision to test mothers and babies was on a case-by-case basis. Others thought that babies could not be drug-tested without the parents’ permission.

**Denise**: I didn’t find out about it until after I asked my doctor, and that was because all my friends were saying “Oh, you need to stop smoking pot, you need to stop doing this, da-da-da-da-da.” And I was like, wait a minute, I walked in my doctor and I asked him, “When the baby’s born, are you doing to test me? Are you going to test me and the baby?” “No, we’re not gonna test you unless you drop dirty at your visits.” And I was like, so wait, when you make us pee in the little jar every time….

**Interviewer**: It’s a drug test every time?

**Denise**: Yes, that’s a drug test every time. And that’s because it’s CPS’s way of knowing if you’re doing drugs beforehand, they’re gonna take that baby instantly from you. It’s only for the hardcore drugs.

**Interviewer**: Do you know if they drug test every baby?

**Sarah**: I don’t know. I would imagine it’s every baby. Because I don’t know why they would single-handedly pick out me, maybe because my knuckles are tattooed [*laughs*]. Well, I mean, [my husband]’s got track marks, you know, they might have saw that and thought that maybe I was doing it, too.

Others were uncertain about the “rules,” like which substances could be detected, which would trigger CPS involvement, and how far back into the pregnancy a meconium test could detect substance use.

**Vicki**: Well, ‘cause my friend Karen says now is a good time to quit cold turkey, because they do check—Sparrow does check the umbilical cord, and… her baby had—but she had different, she had an addiction, like my friend Jamie, like to heroin, but she said if there’s like a certain time limit that if you stop, it won’t show up in your system or whatever. It’s like… see, I didn’t—I don’t know anything about that.

Some uncertainty may be attributable to variation in testing and reporting policies between different obstetric clinics and hospitals. Medical organizations have some discretion in their policy decisions, although they are of course subject to federal and state laws and administrative codes. At the federal level, hospitals must comply with the Keeping Children Safe Act of 2003, which added requirements to the Child Abuse and Treatment Act. Under the act, states are required to develop procedures requiring healthcare providers to notify CPS if they suspect a child has been subjected to drugs, or is suffering from withdrawal symptoms at birth.

Individual clinics and hospitals likely have varying internal policies regarding what is to be detected through urinalysis, along with other testing and reporting procedures. It is unlikely that most women are aware of the numerous federal and state laws and policies. Inquiring about the drug testing policies at a clinic or hospital may increase staff’s suspicion of one’s substance use habits and is unlikely to be of much use, as most women in this study had no choice in the clinic they attended or where they delivered their babies.

### Strategies for avoiding detection

To manage the risk and uncertainty of being identified as a substance-using pregnant woman, women in this study adopted various strategies. Some strategies seemed pro-social and pro-health, like being honest with medical practitioners or seeking out treatment. Other strategies seemed more damaging, like isolating oneself from friends and family who might detect the substance use, hiding or denying the pregnancy, timing prenatal appointments so that persistent substance use would not show up in drug tests, skipping some prenatal visits or avoiding prenatal care altogether.

### Honesty

Six women (27.3%) adhered to the idiom that honesty is the best policy and were up-front with medical practitioners. They felt that being honest showed that they were good mothers despite their substance use and they hoped that doctors and nurses would appreciate their honesty and affirm their motherhood identities:

**Interviewer**: Are you worried about them drug-testing you or anything like that?

**Vicki**: [*emphatic*] Yeah. [*nervous laugh*].

**Interviewer**: Do you still go [to your appointments]?

**Vicki**: Yeah. That’s why I still go, because I want to show them I’m not a big time drug user — if something does happen, I’m not a big-time drug user and… I do care about myself and I do care about the baby’s health, you know. I have a friend who has a baby due any day, or in a week or so, and she hasn’t had no prenatal care through her whole pregnancy.

**Kim:** I mean, I was honest, and I think that’s the best thing, so I was able to tell them “Look, I did smoke marijuana and I didn’t know that I was pregnant, and I’m not addicted, and is there anything you can give me to where it would be out of my system or anything you can tell me about the effects on being pregnant?” I asked for information, and I think a lot of those people respect you a little more, as to where there won’t be so much concern, because if you’re hiding it and they see it right in your levels, especially being pregnant, there’s going to be some concern and they’re going to go behind your back and call CPS. With me being so blunt, so open and wanting the help, I think it shied a lot of people away from being so concerned or disturbed.

In these excerpts from interviews with Vicki, a methamphetamine user, and Kim, who was using alcohol and marijuana, both women express their hope that being up-front with doctors would help them be perceived as good mothers who were concerned about the health of their fetuses, resisting the master narrative of substance-using mothers who are selfish and unconcerned. Vicki was pregnant at the time of her interview and was yet to see if her strategy would be successful. Kim had stopped smoking marijuana before the birth of her daughter and was only using alcohol (albeit heavily), so she did not have any contact with CPS.

Not all women were pleased with the outcome of their strategy to be honest with their doctors. Melinda had been honest with her doctors about her opioid and benzodiazepine use but felt that this strategy had not worked for her, because she was unhappy about how long her son had to stay in the nursery before he could come home with her:

**Melinda**: I would *never* advise somebody to have a child [at the hospital]. I thought I was helping my child by being honest during my pregnancy, I thought I was helping him if I was honest with my doctors. No, I wasn’t. All I did was damage that relationship and our early bonding by letting them have that “in” to keep him from us. We could’ve, and we would have, taken better care of him than what they did, leaving him in his bassinet with a million other babies in there and not enough people to take care of all the babies.

The risk of being honest may be lower when women are using legal or socially-accepted substances or when a woman has a trusting relationship with her medical provider. The relationship between a woman and her medical provider might be one way that socioeconomic status grants some substance-using women privileges and health benefits. If a woman has health insurance and a private doctor with whom she has a long history, honesty may be a safe strategy that allows her to receive support and treatment specific to her risk status. If, in contrast, a woman must rely on a public health clinic that she can attend only when pregnant and where she may see a different doctor every time, she may not know the doctor or the practice’s drug testing and reporting policies and will not have the opportunity to develop a trusting relationship with the practitioner. In this case, revealing one’s identity as a substance-user seems a more risky strategy, because the outcome is more uncertain. These possibilities suggest an area in need of further research.

### Social isolation and denial of pregnancy

Another set of strategies women employed was to keep to themselves, avoiding friends and family who might report them to CPS. For two women, this went as far as concealing or denying their pregnancies:

**Interviewer**: Did you do anything to try and hide it or avoid getting caught?

**Kim**: Yes, I did, I did, um, to hide the pregnancy, I denied that I was pregnant. I drank as if I wasn’t pregnant, and I denied some more, I kept denying. And I lied, a lot.

Of course, pregnancies are typically only concealable for a limited amount of time. Another strategy for women was to socially isolate themselves from anyone who might report them to CPS:

**Interviewer**: Did [fear of detection] stop you from doing anything you might’ve otherwise wanted to do, like stop you from doing something you wanted because you were worried?

**Alice**: Yes, yes. I had stopped talking to everyone, period, because I didn’t want the wrong person to go over there and say something. I didn’t want them to go do that and I didn’t know who to trust, so I wasn’t saying anything.

The strategy of avoiding people may be based on women’s past experiences with CPS. Of twenty-two women who reported having past contact with CPS, the most commonly mentioned source of contact (n = 10, 45.5%) was a report to CPS by a third party. These third parties included roommates and friends, family members, ex-partners, and neighbors. Some of these reports were made out of concern for the children, but many reports were identified by the women as acts of retaliation. For example, a mother would get into an argument with another woman and that woman would report her to CPS in retaliation. In another case, a mother broke up with her abusive boyfriend and, in retaliation, he called CPS and told them she was pregnant and smoking marijuana. Other women had family members who wanted custody of their children and would call CPS very frequently, forcing CPS to investigate every time even though they had found time and time again that the children were happy and healthy. In light of these experiences, women may feel that isolating themselves is an effective strategy for avoiding contact with CPS and law enforcement.

### Avoiding medical care

The most common strategy employed by women afraid of detection was avoidance of medical care (n = 12, 54.5%). This strategy included scheduling visits around their substance use so that any tests would come up negative, skipping some visits, or avoiding prenatal care altogether.

Women who used substances that are only detectable through urinalysis for several days after use were able to schedule their appointments around their substance use.

**Interviewer**: And during this time, while you were pregnant, were you ever worried that if you went to a doctor, they would drug test you?

**Sarah**: Kind of, yeah. Kind of. But that was only a couple of days after I did the heroin. But I would make sure that, um, I would do it on days like, ‘cause you know, that stuff lasts in your system for three to four days, so I would make sure not to do it around the time of the appointment, just to be on the safe side.

**Denise**: I drank a lot of water. I always made sure that I stopped certain stuff before I went in. I had it already charted out for how long it took to get out of my system, this, that and third, like, I made sure I had my stuff on lock. It’s the good thing about being able to make your appointments before you go in.

Some women, like Denise and Amelia, seemed proud of their ability to avoid detection. Amelia laughed, “A lot of people think drugs are dumb or hippies are stupid, but it’s some hard work, man, it’s like chemistry”. Women would “chart out” on a calendar the days that they used and how long it would be before they would test clean and then schedule medical appointments accordingly. By doing so, they were able to avoid positive prenatal drug tests. This method is not effective for avoiding detection at delivery, though, because meconium begins to form in the second trimester of pregnancy and a positive test can indicate substance use a month or longer prior to delivery (Farst et al. 2011). This is an important consideration if meconium testing is triggered only by positive prenatal tests, as women who use substances that pass quickly through the body may successfully evade detection at prenatal appointments and also at delivery. This strategy is less effective if women deliver at a hospital that tests all mothers and/or babies or makes decisions about testing based on other factors, like late prenatal care or the mother’s appearance, demeanor, or history of involvement with CPS.

Women also reported skipping appointments if they had used recently or avoiding care altogether:

**Suzanne**: I wouldn’t go to the doctor’s. I would skip appointments and things, and stretch them out. I always went because, again, CPS will get involved if you don’t go to the doctor’s, so you still have to go, but you know, you didn’t—you just have to stretch it out or go late or delay it or whatever.

**Interviewer**: And did worrying about being involved with CPS or getting her taken away, did it keep you from doing anything you might otherwise do?

**Elsie**: I just didn’t go.

**Interviewer**: Didn’t go to the doctor?

**Elsie**: Yeah. I just wouldn’t show up, I was so scared.

**Alice**: My third child, I had no prenatal care.

**Interviewer**: For what reason?

**Alice**: Because I was taking drugs, well, not drugs-drugs, I was down there smoking on marijuana and drinking liquor. And they told me if they see THC or something like that in my system, then protective services would get involved. So I didn’t go to no care for her, none.

Research repeatedly demonstrates that substance-using women who receive prenatal care experience more positive birth outcomes and have greater opportunities for other health promoting interventions than women who do not receive care (Berenson et al. 1996; El-Mohandes et al., 2003; Green et al. 1979; MacGregor et al. 1989; Racine et al. 1993; Richardson et al. 1999). Prenatal care appointments provide practitioners the opportunity to connect women to needed resources, to screen them for dangerous illnesses or injuries, to screen for intimate partner abuse victimization, and to implement many other public health interventions. By adopting policies that scare women away from treatment, clinics and health organizations lose the opportunity to intervene and promote maternal and infant health.

### Substance abuse treatment experiences

Twenty women (66.7%) had sought substance abuse treatment at some point in the past and had navigated barriers to finding, affording and attending different types of treatment programs. Of the ten women who had not sought treatment, most used only alcohol, tobacco, and/or marijuana. Two of these ten women used methamphetamine, one used assorted prescription pills, and a fourth used hallucinogens.

The twenty women who had experience with substance abuse treatment had explored a variety of different programs, from short-term detox and outpatient support groups to residential treatment and long-term methadone maintenance. Each program type came with its own limitations and barriers to entry.

### Detox

Three women had sought out treatment facilities that would allow them to detox (most commonly from opioids). These programs were very short-term, usually less than a week, and offered medically-assisted or unmedicated detox. Women were in agreement that unmedicated detox was an awful experience and that they would only stay at places that would give them medication to help with their withdrawal symptoms. At some places, such medication was promised but not delivered:

**Tasha**: When I went there, oh my God, [treatment center] was awful. I wouldn't send my dog there. I went there during the day and the lady was really nice. “Oh we'll help you, we'll give you something to ease the withdrawal and help you sleep and we'll keep you comfortable.” I'm like okay, this is what I need, this is where I need to be. And that night, they refused to give me anything to help with the withdrawals and I was freaking out and I was sick and I had just had it. Two o'clock in the morning, I ended up walking out of there. They wouldn't help, they just basically looked at me like I was some horrible drug addict.

**Interviewer**: So you walked out of there?

**Tasha**: Mmhmm [yes], gave up on that and went right back to using.

Even if Tasha had stayed and detoxed, such programs frequently offer little in the way of aftercare unless they are paired with residential or outpatient counseling. Women who had detoxed, with or without medical assistance, reported that the process did nothing to address the triggers for their substance use. They spent up to a week in detox but then returned to the same environment and same social setting they had been in when they were using.

A problem with detox is that it is rarely a possibility for women who are already pregnant. Though the physical withdrawal symptoms are unpleasant for adults, they can be lethal for the fetus. For substance-dependent women who wanted to continue their pregnancies, withdrawal was a dangerous choice, and few medical professionals would agree to supervise the process. Kellie found out that she was pregnant and didn’t want to start taking methadone, so she tried to find a treatment center or a hospital where she could be monitored while she went through withdrawal from heroin. She couldn’t find anyone who would help her:

**Kellie**: It was just the whole, I guess liability issue of the miscarriage associated with treatment and withdrawal of the pregnancy that really scared people. And even when I went to [the local hospital] and said “Can you guys watch me while I detox?” and they said no, I mean, I even—and then they ended up sending me home, and I was like “I’m sick, can you at least send me home with some Vicodin or something?” and they were like, no, so I said “So you’re going to send me home to have a miscarriage, then?” and they ended up writing me, like 10 Vicodin or something.

According to Kellie’s understanding, the medical staff did not want to monitor her withdrawal for fear they would be liable if anything happened to her fetus. Instead, they gave her more opioids to stave off the withdrawal and then turned her away. Kellie continued to use heroin while seeking out other treatment possibilities.

### Opioid replacement therapy

Opioid replacement therapy is the practice of replacing illegal opioids with longer-acting opioids like methadone or buprenorphine administered under medical supervision. Methadone emerged as a treatment solution for heroin addiction in the 1960s. Methadone maintenance therapy spread quickly and became the gold standard for treatment of opioid dependence, “probably the most evaluated form of treatment in the field of drug abuse treatment” (Farrell et al. 1994: 997). It is recognized as the most effective treatment for heroin addiction according to reviews by the Institute of Medicine (1995) and the National Institutes of Health (1998).

Despite such robust evidence of the benefits of methadone maintenance therapy, it remains, for some, a highly controversial practice. Since their beginning, methadone programs have been accused of merely substituting one drug for another (Joseph et al. 2000). Methadone maintenance programs have been cited as an example of evidence-based medical programs that have been adversely impacted by misperceptions and biases, limiting their implementation and reach (Gordis, 1991). As a result, patients fear that the stigma associated with being a methadone user will negatively impact their jobs, their social relationships and the medical care they receive (Joseph, 1995). Stigma and discrimination appear to be powerful forces preventing the full acceptance of methadone treatment, and likely impacts both pregnant and non-pregnant women seeking treatment.

The controversy surrounding methadone maintenance was demonstrated by women in the current study. Eleven women had, at some point in their lives, sought opioid replacement therapy with methadone or buprenorphine, another partial opioid agonist more recently approved for opioid addiction treatment and known by common product names like Suboxone and Subutex (FDA, 2013). Although most women were overwhelmingly in favor of opioid replacement therapy, many of the same women were concerned about never being able to stop taking methadone.

**Alyssa**: I would honestly say it’s the day I got on methadone, because it totally, has totally changed my life, because as an adult, you know, I really didn’t lead the greatest lifestyle up until the last two years, and prior to that I don’t have any good memories, so. […] I had a lot of people say “Methadone’s like liquid handcuffs.” You still have to get it every day. I look at it as you’re not going out and getting into trouble, but some people still look at it as you have to have it, and you have to have it every day or else you’re sick, so it’s a… you know, now I look at it different, I’m glad it was there to change my life.

Others were less effusively appreciative of methadone treatment but still felt that they could not have achieved sobriety without it:

**Eleanor**: I needed something – no. I mean, I wish I didn’t need, didn’t need to get on the methadone, I wish that I would’ve been able to do it the other way, you know, without any medication, but no, I wouldn’t say it makes me weak. I would just—you know, I didn’t need some help, but when I got on it, I was able to do it. Because there are some people on methadone that still use, and continue to use, and even that doesn’t… doesn’t help them. So you know, with a little help I was able to pretty much beat my addiction.

Most women shared similar experiences, but two women expressed a strong dislike for methadone maintenance. One woman called methadone “liquid handcuffs,” because she felt that once someone started taking methadone, they would be on it for life. Naomi explicitly described many of the arguments made against opioid replacement. She had recently used Suboxone (buprenorphine) to recover from her dependence on opioid painkillers but had made a point to wean herself from it quickly thereafter:

**Naomi**: I went to [a residential treatment facility] and… I forgot what they gave me, it wasn’t methadone… Suboxone.

**Interviewer**: Suboxone?

**Naomi**: Yep. And I got Suboxone, and I’ve been clean since.

**Interviewer**: So you got the Suboxone at [treatment facility]?

**Naomi**: Yep. I was in their detox facility for three days, and then I went into their residential program.

**Interviewer**: Are you still on the Suboxone?

**Naomi**: Nope. I think [Suboxone maintenance] is retarded [*laughs*]. All it is is a legal way for you to get high. Most people abuse it, they don’t take it the way they’re supposed to. […] You’re still getting high, and you’re not going through withdrawal. All it is is a state-funded way for you to get high. Now the state’s paying for your way to get high, and that’s the way I feel about methadone.

Women on maintenance programs were aware of these perceptions of their treatment programs and explained why “substituting” opioids was so successful:

**Loretta:** And people are all “Well, you’re substituting for another drug” and da-dada-dada, well, the reason why it works is because it’s legal, so you change your whole lifestyle. Because of the fact of it being legal, you don’t have to deal with the illegal aspect of taking it and dealing with all the illegal people and all the stealing and doing this to get it and doing that to get it and running around with drugs on you. You don’t have to do that no more, ‘cause it’s legal, so you don’t have to be in that whole circle no more, and you get yourself away from places, people and things, and it works.

As with other treatment options, women encountered barriers to enrolling in methadone programs. Interestingly, the barriers they encountered were the opposite of what one might expect. Women who were pregnant were able to enroll in programs immediately:

**Interviewer**: How was your experience trying to get into [the methadone clinic]?

**Cora**: It was really easy, because I was pregnant, so I got on the same day. But if I wasn’t pregnant then it takes a couple weeks, so you have to use, and so on and so forth.

Women who sought out methadone maintenance treatment when they were pregnant had no difficulty enrolling in a clinic. Women who were not pregnant when seeking treatment were not so successful. Brittany had unsuccessfully sought methadone treatment after the birth of her second son and had not been able to overcome the barriers she encountered. She continued to use and became pregnant again, and finally lost custody of all three of her children.

**Brittany**: I think, I think the program has changed a lot, though, honestly, because in between the time when I – after I had [my second son], like a couple of months, we really briefly tried looking into going to another one at that time… I don’t know if like the requirements changed or something, but it was, it was a different point, too, though, but it was a lot more running around and we never ended up going through with it. ‘Cause it was like, well, first they wanted us to go see a doctor, and they wouldn’t take our insurance, because they wanted us to take a heart test first… and then it was just like, so much drama with that that we never ended up going through with it.

**Interviewer**: So there was a lot of messing around?

**Brittany**: Yeah, like I said, I don’t know if the requirements changed, ‘cause it was a couple of years before when I started coming to this one and it was a different clinic, too, so maybe they had different requirements, but it was where it was so ridiculous in the end that we didn’t go through with it, whereas with this one, if you have your money for the week, you show up that day and you’re basically starting that day. Whereas the other one it was different, it was like they wanted us to wait a couple of weeks in between, you know. And you have like a fleeting moment between when you have the money in your hand and you wanna start [treatment and] when you start shutting down, so…

Once enrolled in methadone programs, women were concerned about continuing to pay their bills. Women who were pregnant or who had recently given birth were eligible for Medicaid, which would cover the cost of treatment, but they worried about what would happen to them once they no longer had insurance:

**Alyssa**: With the methadone, I do have my Medicaid that pays for it, and I do sometimes worry like, “What if that gets cut off?” Because it’s expensive. But I would just have to find – I would have to find a way to pay for it. But, I mean, it’s… the community has been pretty good in helping find, you know, helping me find the help that I need to get clean.

Other women were cobbling together some Medicaid allowances and assorted grants, but were facing the possibility of being rapidly tapered off methadone if they could not afford to continue paying for it:

**Melinda**: I got a grant to go to [a treatment program], grant funding, and then I somehow got Medicaid to help with the methadone treatment, however, that may be in jeopardy, and if they’re not going to pay for it, I’m gonna have to get off of it a lot faster than would be healthy, and I’m scared. […] I’m just gonna be screwed, and then there’s gonna be no way out unless I use heroin or something to survive. I don’t know. I’m scared of that possibility.

Finally, women who did take methadone during their pregnancies felt that there was insufficient information about what they should expect at the hospital and when they brought their infants home. Methadone has been deemed safe for use during pregnancy but can still produce symptoms of withdrawal in exposed infants. Some women were surprised at the severity of their infants’ withdrawal symptoms:

**Alyssa**: But man, having my daughter, being on methadone, I know it changed my life, but shoot, I went and got my tubes tied. That’s how much that methadone—I don’t understand how women can have child after child on the methadone.

**Interviewer**: Really? That bad, huh?

**Alyssa**: Watching my daughter go through it? Yeah, that bad. It really woke me up, I want to come off methadone, I’m at that point, you know, yeah. It wasn’t fair, it wasn’t fair to her. I don’t think my doctors were 100% honest, you know, I was already on the methadone when I got pregnant, so there was absolutely nothing I could do, but, you know, they sugar-coated it. We were in the hospital five weeks, she was on a very high dose of morphine, and she had to be on phenobarbital and just, it sucked. And still, if you get loud and go up to her, you’ll startle her, and she’s just now getting on a normal sleeping pattern and, yeah, it’s hard thing—it’s hard to watch your child go through that, knowing it’s something you did, you know.

Others were unprepared for how they would be treated at the hospital. In some cases, they were informed by medical personnel that CPS was called for all mothers using methadone, whether it was prescribed or not. Others reported that CPS was mistakenly called. Kellie felt trapped by hospital policies about methadone use, as she thought that enrolling in the methadone clinic would help her escape involvement with CPS:

**Kellie**: I guess I feel kind of confused as to, they tell you [methadone treatment]’s your only option yet it’s considered so questionable or harmful that they have to call CPS, it’s required for your baby and stuff.

Kellie had used other opioids and marijuana throughout her pregnancy, so it is possible that CPS was called because of the presence of those substances in her son’s meconium and that she misunderstood the hospital’s policy. However, she was not the only woman confused about, on the one hand, methadone as a prescribed treatment and, on the other, its role in their newborns’ withdrawal symptoms and their involvement with CPS. The confusion was shared by other women in the study and by the public health nurses with the county health department’s Family Outreach Services, who were in the process of trying to assemble articles and pamphlets about methadone use during pregnancy so that they can better prepare the women on their caseloads for their experiences with hospital delivery, watching their newborns’ withdrawal symptoms, and soothing their babies when they go home.

### Residential treatment

Fifteen women, half of all women in the study, had experience with residential or in-patient treatment programs. These ranged from general “rehabs” to special programs in prisons. Natalie said that the most effective treatment she had ever received was an inpatient program inside a women’s prison. She called it “RSAT,” which may be one of the Residential Substance Abuse Treatment programs administered through the Bureau of Justice Assistance (2012).

**Natalie**: And the last six months of my prison sentence I did a program called RSAT and it’s an inpatient treatment inside the prison […] I went through all that and I started bringing them emotions out and digging deep and talking about what I went through and stuff, I had to look at it and deal with it. And a lot of that has so much anger in me, anger towards my mom, towards the court system, towards everybody that failed me all my life, as a child. And then, you know, anger at myself with losing [custody]. Once I dealt with all of that, it really, really changed who I was inside and it made me stronger. You would think I had a lot of strength going through everything I went through, but I just buried everything under drugs.

Natalie had been in other residential programs before RSAT and had not found them effective. After leaving prison, she did return to substance use briefly before desisting for some time. At the time of her interview, she reported that she had relapsed for a few months at the beginning of the current year and became pregnant at the end of that period, and now she felt that she would be clean for good.

Hazel had been to a residential treatment program to help her overcome her addiction to crack cocaine. She found the classes offered there very helpful, both in their instruction but also for the social opportunities:

**Hazel**: Well, the classes helped, too. They had classes in the rehab, the lifestyle changes class, different classes I could take. The, um, I say the lifestyles class is the one that helped me more, because they helped me to prepare for what the real life was really all about, and beside the drugs and all that, I was actually somebody else.

Women felt that residential treatment was not effective if it was too short or there was no outpatient support. Those women who left residential programs and returned to substance-using social networks and environments also returned to substance use. Alyssa recalled finishing three months in treatment and being picked up at the facility by her mother, who drove them straight to the “dope house”. Elizabeth had recently spent two weeks in treatment but was not optimistic about the future:

**Elizabeth**: Yeah, but once I left – I just recently left – once I left, I’m just back out here in the real world.

**Interviewer**: When were you there?

**Elizabeth**: I went on the 21^st^, I think.

**Interviewer**: Oh, wow, you were just there. So after release?

**Elizabeth**: I started back drinking. Haven’t found any cocaine, or it hasn’t been an urge right now. I’ve popped Vicodins.

**Interviewer**: So the real world is not the same as the treatment world?

**Elizabeth**: No. Lot of temptation. […] It’s not long enough, I don’t think so either. It’s not, it’s so not long enough. It’s like a vacation from the real world.

In interviews with women who had sought residential treatment during their pregnancies, references to the same treatment facility repeatedly arose. It became obvious that women were talking about this single treatment facility because, to their knowledge, it is the *only* residential substance abuse treatment program in the state that will accept pregnant women.

**Kellie**: [The nurses at the local hospital] gave me the list of methadone clinics in the area, there’s a couple, and some other rehabs in the area, rehab clinics, and I called all of them on the sheet. None of them would accept pregnant women unless I was already detoxed or on a methadone maintenance. None of them would do a withdrawal while I was pregnant, until I finally found *one* place that was in [distant town], it’s called [name of program]. And they are, as far as I know, the only place in the state that will take pregnant women who are, you know, addicted to opiates and have to go through withdrawal or be put on methadone or whatnot. But unfortunately I did not find them until I was probably about seven and a half months along…

The facility women mentioned is located 104 miles from the study site. At this location, there is an option for children to stay at the facility with their mothers. Childcare during treatment has previously been identified as a barrier to care for substance-using mothers (Blume, 1990; Center for Substance Abuse Treatment, 1994; Finklestein, 1994; Marsh et al. 2000), but women in this study reflected that having their children there was not necessarily helpful:

**Cora**: Yeah, I went somewhere where I could take my kids, and I ended up taking my youngest, and she ended up getting abused by other children in there that had it way worse than my kids had it. They had no training at all, they didn’t have contact, they were almost feral. […] I think it’s the only place in Michigan. And I don’t really think it should be a setting—I mean, it’s good for some people, but you can’t concentrate when you gotta go everywhere with screaming kids in recovery, you just can’t.

Cora ended up sending her daughter to live with her daughter’s father, but for women lacking that option, it is not clear what the solution might be. Cora’s experience suggests that although allowing children to stay with their mothers at treatment facilities may reduce barriers to care for some women, it may reduce treatment effectiveness for others.

## Discussion and conclusions

The current analysis provides an overview of the issues substance-using mothers encounter when negotiating prenatal care, hospital delivery, and seeking substance abuse treatment. Women discussed the strategies they employed to avoid being detected as substance-users or, in some cases, explained why they had not feared detection. Women who used alcohol and tobacco were less likely to fear being identified by medical professionals or law enforcement authorities than women who were using illicit substances. Some women who were using illicit substances were not afraid because they had no personal or vicarious experience with the consequences of detection, particularly loss of custody. Of the women who did fear detection, some were up-front and honest with their doctors, and they felt that this would protect them from the worst consequences because their doctors and nurses would appreciate their honesty. Others hid or denied their pregnancies, isolated themselves away from others who might report them to authorities, and delayed or avoided prenatal care.

The results suggest that punitive policies have indeed had some chilling effect on women’s help-seeking behavior by discouraging women from accessing prenatal care or leading them to skip appointments, and by motivating women who did attend appointments to withhold medically relevant information about their substance use. Some women were honest with medical professionals but then experienced poor treatment, making them less likely to be honest again in the future. Women also shared their experiences accessing substance use treatment. The benefits and drawbacks of different treatment options were discussed, as well as the barriers women encountered as they searched for and received treatment.

These findings demonstrate that women are in need of more treatment options, better access to the treatment of their choice, and more support for staying in treatment. The women in this study revealed that in their searches for residential treatment centers they could locate only *one* facility that would accept pregnant women or women who needed to bring their children with them. This treatment facility is located more than a hundred miles from the study site, making transportation and visitation expensive and time-consuming. Women would benefit from an increased number of residential care facilities. There are several methadone clinics in the study area and women who sought treatment there when pregnant were pleased to find that their status as pregnant women afford them expedited enrollment in treatment. This is an excellent policy that should be continued, as most women spoke positively about their experiences on methadone. However, when women sought methadone treatment *between* pregnancies, they faced waiting periods of days or weeks. During this delay, women continued to engage in risky substance use and, in some cases, lost their desire to enter treatment. Increased funding for methadone treatment clinics to support larger client populations would help to cut down on these waiting periods and get women into treatment when they are motivated to enroll. Additionally, increased grant funding to help women stay in treatment once they are enrolled would help to decrease women’s anxiety about what will happen if they can no longer afford to pay for their methadone.

Women’s experiences seeking methadone treatment also highlighted a need for more information about this treatment option, both in general and specifically for pregnant women. In general, women harbored some misconceptions about methadone and were unclear about the treatment process. They were concerned that if they start taking methadone, they would never be able to stop. Women who were pregnant and on methadone were not well-informed about what to expect when their babies were born. They did not expect to see such severe withdrawal symptoms, they did not know that CPS would be called in for hospital and home visits, and they did not understand the way doctors and nurses assessed their infants’ withdrawal symptoms and administered treatments. This lack of information left women feeling confused, vulnerable and in some cases misled or betrayed by treatment professionals. Better communication between medical staff and mothers may help to ease some of this confusion and reduce feelings of stigmatization and unfair treatment. Methadone clinics should offer information sessions and materials to help prepare pregnant women for the experience of delivering their babies at hospitals, including what to expect in regard to pain management, infant withdrawal symptoms, CPS involvement, treatment approaches for withdrawing infants, and how to work with doctors and nurses to help the process go smoothly. These information sessions could also include advice for comforting methadone-exposed babies once they come home.

A major implication is that women would benefit from some sort of wrap-around or comprehensive care and professional advocacy. The few women in the study who were working with public health nurses were appreciative of the way the nurses were available to answer their questions, help them with transportation to and from appointments, and help them access resources like car seats, cribs, baby clothes, and childcare assistance. Expanding similar programs to increase enrollment and funding support would likely be of great benefit to women in similar situations. Public health nurses could be important advocates in court cases, as they can attest to women’s efforts to desist from substance use and perform their motherhood responsibilities. Home-visitation nursing programs show great return on investment for at-risk populations (Eckenrode et al. 2010; Karoly et al. 1998; Karoly 2005; Kitzman et al. 2010; Olds et al. 2010) and benefit not only the direct recipient, but demonstrate spillover effects for other families and the community (Karoly 2005). Such programs should be considered a very strong policy option for pregnant women and mothers struggling with substance use.

The findings of this small, exploratory study have important limitations. The current research presents the perspectives of substance-using mothers. They expressed frustration and anger with the system, which included treatment professions, CPS caseworkers, judges, attorneys, social service providers and law enforcement. In many cases, women’s anger and frustration with the system seemed justified, but there is a need for a better understanding of the perspectives of individuals on the “other side” of this social problem. Future research should also incorporate the perspectives of medical professionals, CPS caseworkers, and members of the court system to develop a more complete picture of how the system functions and how women’s frustration and anger might be reduced.

Substance use during pregnancy and motherhood is an emotionally-charged social problem in need of a compassionate and evidence-based solution. A greater effort should be made to incorporate women’s voices, as they are the authorities on their experiences. Their perceptions of barriers to care and the types of treatment they receive have important implications for their likelihood of compliance with treatment and potential desistance from substance use. This study provided an outlet for their voices and has identified promising avenues for future research and policy development. Future research should continue in this direction with the goal of improving maternal and infant health outcomes for this population.
